# Supplementary material for: Unraveling a tumor type-specific regulatory core underlying E2F1-mediated epithelial-mesenchymal transition to predict receptor protein signatures
Source: Nat Commun. 2017 Aug 4;8:198. doi: 10.1038/s41467-017-00268-2 (PMC5543083; doi:10.1038/s41467-017-00268-2)
Supplement: Supplementary file 1 — Supplementary Information [file 41467_2017_268_MOESM1_ESM.pdf]

File name: Supplementary Information
Description: Supplementary figures, supplementary tables, supplementary methods and supplementary references.

File name: Supplementary Data 1
Description: Feedback loops (FBL) in E2F1 interaction map, Structural and biomedical properties of each feedback loop in bladder and breast cancer. Supplementary Data 1 contains five sheets including three nodes feedback loops identified in E2F1 interaction map; ranking score of in bladder and breast cancer along with top ranked FBLs in both the cancer types.

File name: Supplementary Data 2
Description: Logic-based rules for bladder (sheet 1) and breast (sheet 2) cancer regulatory core simulations.

File name: Supplementary Data 3
Description: E2F1 map interactions (CellDesigner version).

File name: Supplementary Data 4
Description: Interactions between nodes in E2F1 map (Cytoscape version)

File name: Supplementary Data 5
Description: Effect on EMT phenotype after single/ double perturbation simulation experiments in bladder and breast cancer.

File name: Supplementary Data 6
Description: Analysis of Steinway's EMT map.

File name: Supplementary Data 7
Description: Pathological stages associated with predicted (sheet1) and randomly generated (sheets 2-31) molecular signatures in TCGA bladder cancer cohort.

File name: Supplementary Data 8
Description: Pathological stages associated with predicted (sheet1) and randomly generated (sheets 2-31) molecular signatures in TCGA breast cancer cohort.

File name: Peer review file
Description:

Supplementary Methods

Network analysis

To evaluate the structural properties of the E2F1 regulatory network, we converted the E2F1 map into a format suitable for analytical tools such as Cytoscape[1]. Towards this, all types of reactions were categorized into activation, inhibition and neutral interaction (Supplementary Data 4). Moreover, complexes that take part in a reaction were dissected and separate reactions were established for their components, for example: a reaction for complex 'AB' that activates 'C' was split into two separate interactions: (i) 'A' activates 'C'; and (ii) 'B' activates 'C'. The purpose to dissect the complexes into separate reactions was to map the post-transcriptional regulatory layer (microRNAs) and expression data for the identification of the core regulatory network.

Topological properties of the map

The Cytoscape version of the E2F1 molecular interaction map contains 1015 nodes and 4180 interactions. We calculated all the topological properties of the network using Cytoscape plugin 'NetworkAnalyzer' (Supplementary Table 1). The average number of neighbors for each node in the network is 7.89, which indicates that the network is well-connected. We calculated the average clustering coefficient of the network ($\bar{C} = 0.226$) and the network diameter ($D = 8$). The clustering coefficient indicates the density of connections among the neighbors of a node[2]. The comparably large value of $\bar{C}$ and the large diameter of the network indicate the modular organization of nodes in the network[3,4]. Furthermore, we fitted a power law of the form $y = a + x^{-\gamma}$ to the clustering coefficient distribution. The results ($a = 0.906$; $\gamma = 0.710$) indicate a hierarchical structure of the network[4,5]. The small average characteristic path length ($l_G = 3.258$) and large average clustering coefficient indicate that the network has a small world architecture[2,5,6]. The small world property reveals that signals can propagate very fast through the whole network. The values of important topological properties for each node are shown in Supplementary Data 4.

Some of the node properties (node degree and betweenness centrality) of the E2F1 interaction map were mapped to visual properties in Supplementary Figure 1. In this representation the node size is determined by its degree, i.e. number of edges connected to the node and node color

denotes the betweenness centrality (green: low; red: high), i.e. the amount of control that a node exerts over the interactions of other nodes in the network. As the network was constructed by focusing on interactions around E2F1, it is no surprise that E2F1 represents the largest node followed by other members of the E2F family (E2F2 and -3). Other nodes with very high node degree are TP53 and MYC, which are known for their delicate role in tumorigenesis. E2F1 has also the highest betweenness centrality value ($C_b(E2F1) = 0.4222$) indicating that E2F1 plays a central role for the signal flow in the network. By determining these topological properties one can identify important nodes as potential candidates for therapy design[5,7].

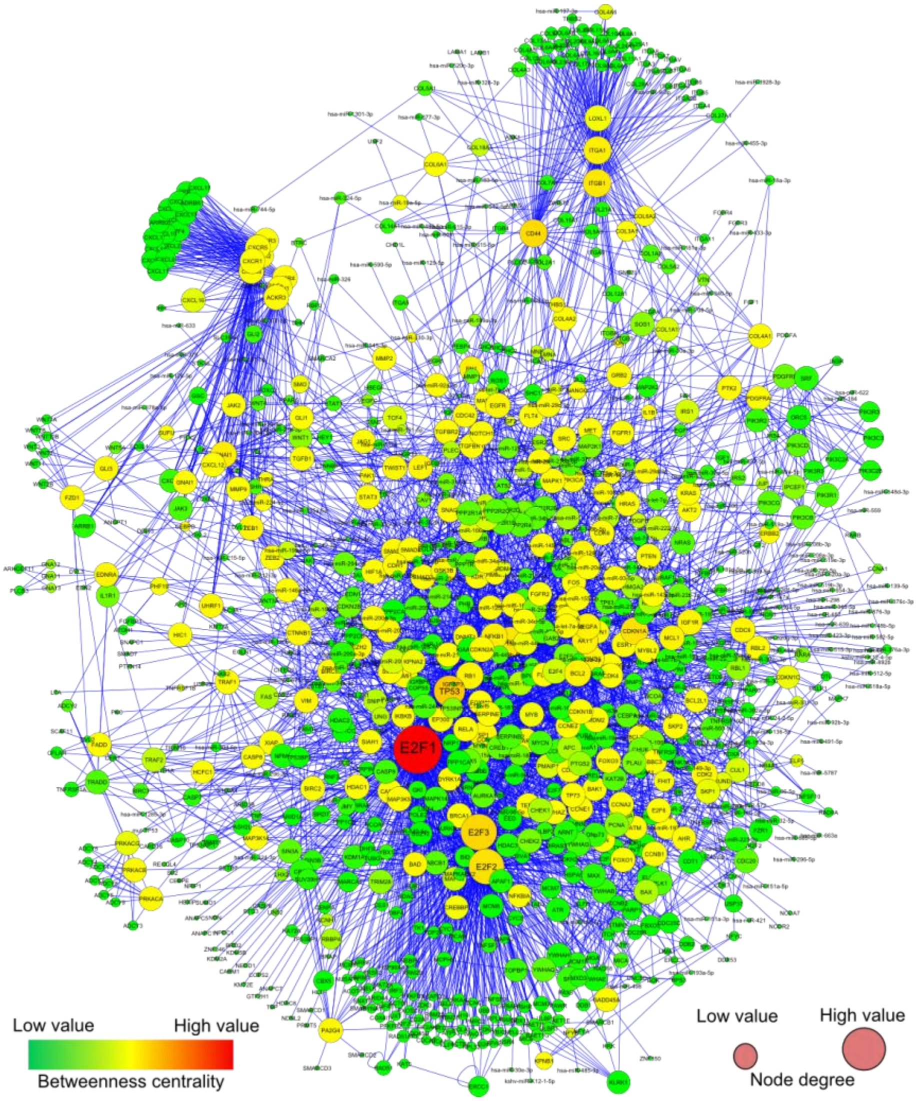

Supplementary Figure 1: Cytoscape view of E2F1 interaction map. The size of the nodes represents the value of the node degree. The node color ranges from green (low betweenness centrality) to red (high betweenness centrality).

Further, we fitted a power law of the form $y = a + x^{-b}$ to the degree distribution of the network nodes (Supplementary Figure 2), where y indicates the number of nodes that share a particular degree x ($a = 320.15$ and $b = 1.249$). From this result, we conclude that the network has a scale-free topology, which is consistent with the fact that the network contains few high-degree nodes also known as hubs. Networks containing few hubs are generally heterogeneous in terms of node degree and are considered to be robust against single random perturbation[5,8].

Supplementary Table 1: Topological parameter values of the E2F1 regulatory network.

**Topological parameter**	**Value**
Number of nodes	1015
Number of edges	4174
Clustering coefficient	0.226
Network diameter	8
Network radius	4
Characteristic path length	3.258
Avg. number of neighbors	7.892

A scalable web version of the E2F1 interaction map in standard SBML format is accessible at https://navicell.curie.fr/pages/maps_e2f1.html. It allows an easy navigation and visualization of the map from an abstract level to a more detailed molecular interaction level.

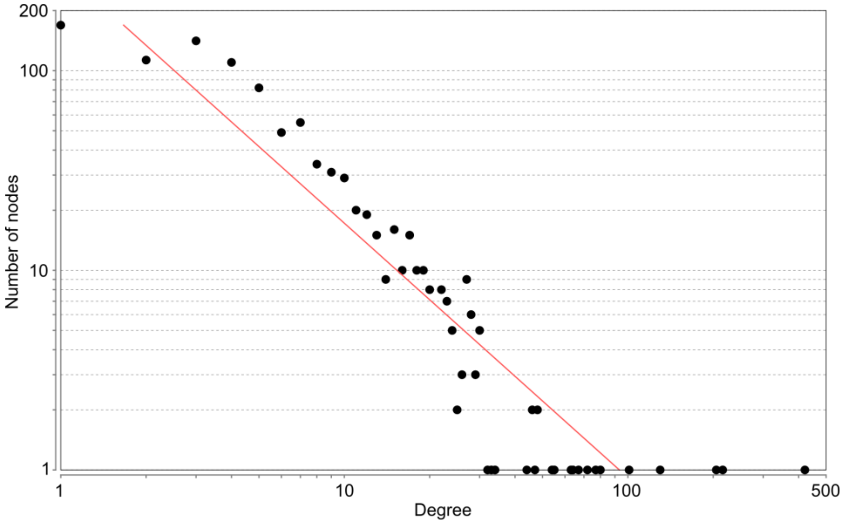

Supplementary Figure 2: Node degree distribution of E2F1 interaction map. The red line indicates that node degree distribution follows power law, which indicates a scale-free topology of the network.

Motif prioritization

We prioritized network motifs based on various structural and biomedical criteria using a multi-criteria optimization function in equation (1):

$$S_{ij} = \frac{w_{1j}}{2} \cdot \frac{\langle ND \rangle_i}{\max(ND)} + \frac{w_{1j}}{2} \cdot \frac{\langle BC \rangle_i}{\max(BC)} + w_{2j} \cdot \frac{\langle DP \rangle_i}{\max(DP)} + w_{3j} \cdot \frac{\langle GP \rangle_i}{\max(GP)} + w_{4j} \cdot \frac{\langle |FC| \rangle_i}{\max(|FC|)} \qquad \text{(Eq. 1)}$$

Here S_{ij} is the ranking score of each motif (i = 1…n) in different weighting scenarios (j = 1…13) as given in Supplementary Table 2. w_{1j} *to* w_{4j} are weighting factors pounding the importance of the chosen properties, $\langle ND \rangle_i$: average node degree, $\langle BC \rangle_i$: average betweenness centrality, $\langle DP \rangle_i$: number of nodes of a motif involved in disease pathways, $\langle GP \rangle_i$: average gene prioritization score, and $\langle |FC| \rangle_i$: average absolute expression fold change of a motif i.

Weighting scheme

We chose five different sets of weighting scenarios, each giving more importance to one or another parameter in Eq. 1. The weighting scenarios are shown in Supplementary Table 2. In total, we used 13 different weighting scenarios for scoring motifs. In the first set, only one parameter was given importance for ranking. In the sets 2-4, we considered two, three and four parameters respectively, and applied consistently higher weights to the absolute expression fold change of the motif to identify tumor type/process-specific top ranked motifs. In the last set, we assigned equal weights to all the parameters considered. The idea behind these different weighting scenarios was to remove any biasness associated with the parameters used in a multi-objective function during motif prioritization.

Supplementary Table 2: Weighting scenarios for motif ranking.

Sets	w_1	w_2	w_3	w_4
Set 1	1	0	0	0
	0	0	1	0
	0	0	0	1
Set 2	1/4	0	0	3/4
	0	1/4	0	3/4
	0	0	1/4	3/4
Set 3	1/8	1/8	0	3/4
	1/8	0	1/8	3/4
	0	1/8	1/8	3/4
Set 4	1/16	1/16	1/8	3/4
	1/16	1/8	1/16	3/4
	1/8	1/16	1/16	3/4
Set 5	1/4	1/4	1/4	1/4

Multi-objective optimization

In multi-objective optimization, one tries to find the so-called Pareto set, a set of non-dominated solutions. The idea behind this is that, if one has several objective functions (e.g. F_1, F_2… F_n) to be optimized at the same time, the non-dominated solutions are those that are not outperformed by any other solution in all the functions considered at the same time. For example, let us suppose we are maximizing F_1 and F_2, and a solution is given by the vector with function values [F_1, F_2]. A Pareto set could be composed by the three solutions with values [100, 0], [0,100] and [50, 50]. Clearly, none of the solutions is better than the others for both F_1 and F_2 at the same time. One strategy to obtain the Pareto set is to merge the functions in a unique objective function by summing and weighting each of them. Here:

$$F = c_1 \bullet F_1 + c_2 \bullet F_2$$

Then the problem becomes to maximize the obtained weighted function:

$Max(F) = c_1 \bullet F_1 + c_2 \bullet F_2$

One can iteratively modify the values of c_1 and c_2 and maximize the problem for each set of weighting factor values. The Pareto set is obtained by merging all the non-identical solutions in terms of the optimization parameters. Supposed in our case the optimization parameters are P = [P_1, P_2, P_3], which influence the values of F_1 and F_2. We now iteratively change the values of the weighting factors c_1 and c_2

$c_1 = 1, c_2 = 0$ => max = $F[1 \bullet F_1 + 0 \bullet F_2]$ => P = [1,2,3]
$c_1 = 0.9, c_2 = 0.1$ => max = $F[0.9 \bullet F_1 + 0.1 \bullet F_2]$ => P = [1,2,3]
$c_1 = 0.5, c_2 = 0.5$ => max = $F[0.5 \bullet F_1 + 0.5 \bullet F_2]$ => P = [4,1,5]
$c_1 = 0, c_2 = 1$ => max = $F[0 \bullet F_1 + 1 \bullet F_2]$ => P = [0,0,1]

In this case, the Pareto set would be the set of non-identical solutions in terms of the optimization parameters P, that is:

Pareto set = {[1,2,3], [4,1,5], [0,0,1]}

Of note, P = [1,2,3] appears twice but we are only interested in the set of non-identical solutions, so we represent it only once in the Pareto set.

We have translated and adapted the idea behind multi-objective optimization to our workflow for selecting the key network motifs based on multiple network properties and cancer associated features. The objective function defined is shown in equation 1 above.

The workflow is as follows:

For each weighting scenario shown in Supplementary Table 2:

1. We calculate the objective function for each network motif
2. We rank the motifs according to the value of the objective function
3. We select top 10 high score motifs

Finally, we select non-identical network motifs that are later used to construct the core regulatory network (see Methods in the manuscript).

Validation of the workflow using another network associated with EMT

To validate our workflow for deriving a regulatory core driving a phenotype e.g. EMT and prediction of molecular signatures, we selected a TGFB1 signaling network developed by Steinway and colleagues[9]. The TGFB1 pathway has a well-defined role in EMT regulation and is dysregulated in a large number of cancer types. Using our methodology as shown in Fig 8, we derived a small regulatory core (Supplementary Figure 3).

Derivation of the regulatory core

We converted the network into a format suitable for structural analysis in Cytoscape and resolved all complexes into single node interactions. Further, we mapped fold change expression data of non-invasive (RT-4) to invasive (UM-UC-3) cell lines of bladder cancer. We obtained a network containing 87 nodes and 236 interactions and determined node degree and betweenness centrality using network analyses. In total we identified 21 feedback loops (FBLs) of three nodes. Using our multi-optimization function (Eq. 1), we ranked all FBLs (Supplementary Data 6) and selected top five FBLs from each scenario to obtain the regulatory core for this network (Supplementary Figure 3).

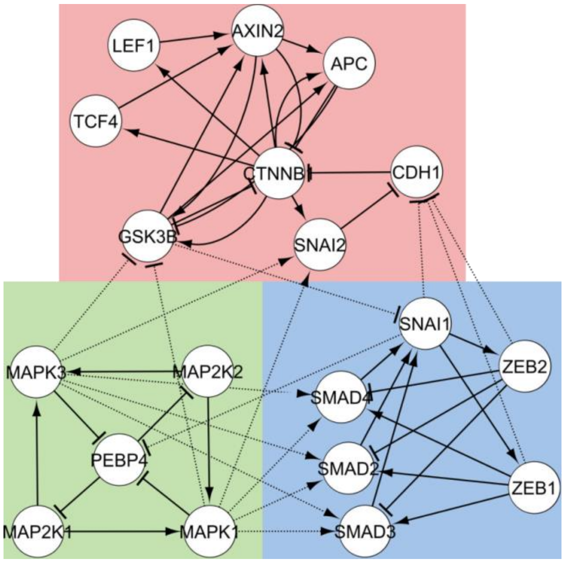

Supplementary Figure 3: EMT driving regulatory core from TGFB1 signaling network derived by Steinway *et al.*[9] using bladder cancer data. The regulatory core contains three disjoint subnetworks (shown in blue, pink and green background colors) which were connected using direct interactions extracted from the TGFB1 signaling network (dotted lines).

A logic-based model for predicting molecular signatures for EMT

We constructed a logic-based model of the identified regulatory core (Supplementary Data 6). The model contains three layers: (1) An input layer containing TGFBR, FZD, RAS and PTCH, (2) a regulatory layer and (3) an output layer which represents EMT phenotype in three ordinal levels from 0 to 2 based on the sum of Boolean states of directly connected factors (Supplementary Figure 4).

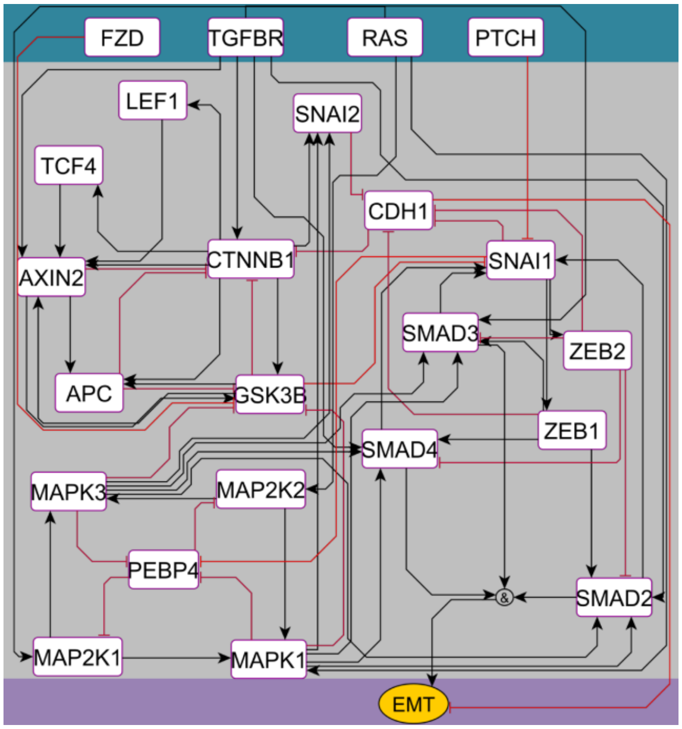

Supplementary Figure 4: Logic-based model of the core regulatory network driving invasive phenotype in bladder cancer. The black and red lines represent the type of interactions (i.e. activation and inactivation). The model is divided into the input layer (blue), the regulatory layer (gray), and the output layer (violet).

Model simulations

Our model simulation recapitulates the epithelial state (EMT=0) when the TGFB1 receptor is inactive (i.e. 0) while PTCH is active (i.e. 1) see Supplementary Table 3. Upon activation of TGFB1 receptor and inactivation of PTCH, the model reproduces the mesenchymal state of a cell (EMT=2). Our model simulations are in agreement with the findings proposed by Steinway *et al.*[9] which support the validity of our methodology.

Bladder cancer				
TGFBR	FZD	RAS	PTCH	EMT
0	0	0/1	1	0
0	0/1	0/1	0	1
1	0/1	0/1	1	1
1	0/1	0/1	0	2

Supplementary Table 3: The effect of TGFBR, FZD, RAS and PTCH on EMT phenotype in bladder cancer. Active state of the molecule is represented by ‘1’, the inactive state by ‘0’. The phenotypic output (EMT) can take three ordinal levels ranging from ‘0’ (non-invasive) to ‘2’ (highly invasive).

Correlation of E2F1 expression and invasive phenotype

Highly invasive phenotype of cancer cells is correlated with high E2F1 expression as shown in case of CCLE data of bladder and breast cancer cell lines as well as in other types of cancers (Supplementary Figures 5, 6).

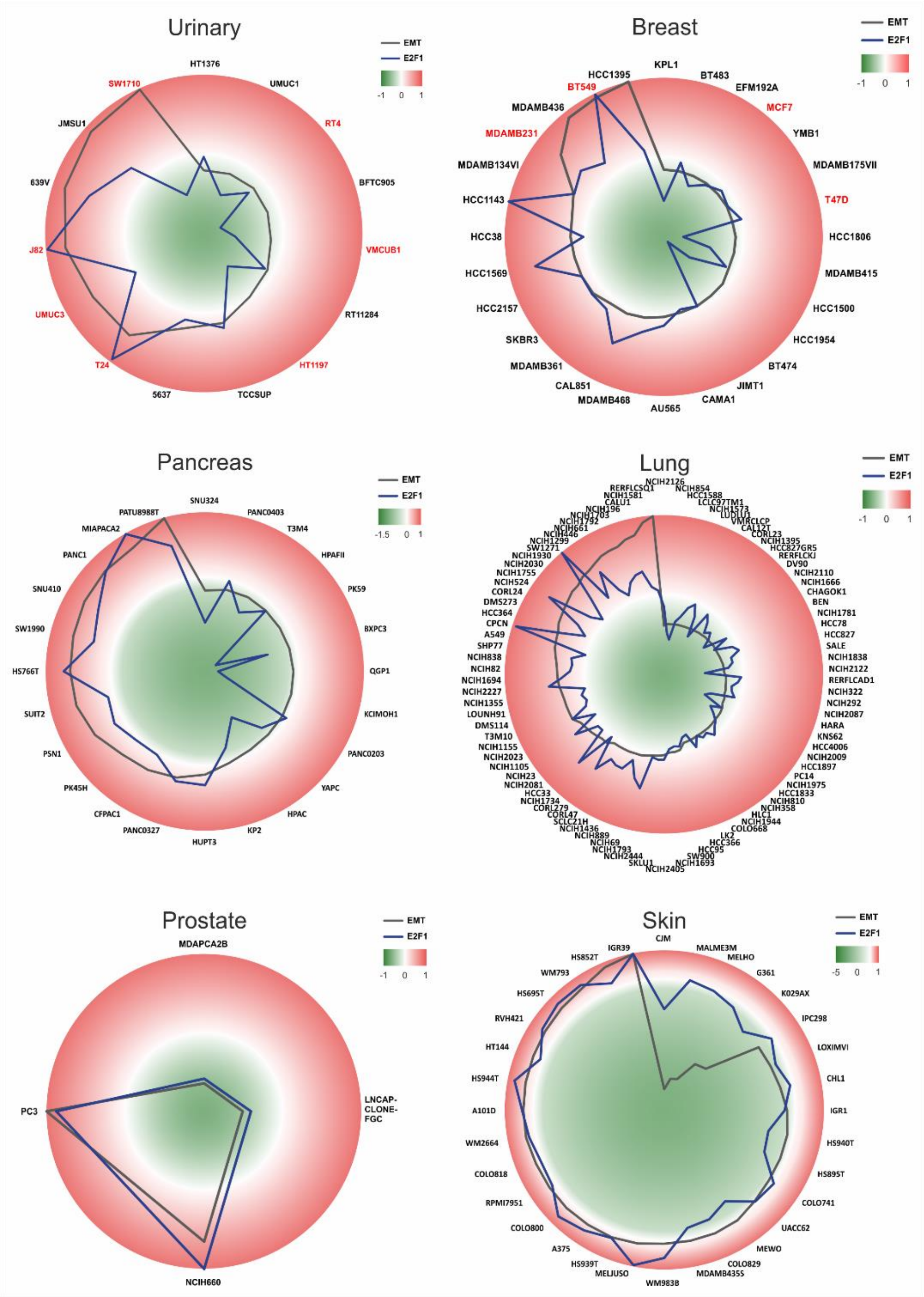

Supplementary Figure 5: Correlation between the expression of E2F1 and EMT markers in various aggressive cancer types. Normalized, median centered mRNA expression data from CCLE database showing that

E2F1 levels in large subsets of bladder, breast, pancreatic, lung, prostate and skin cancer cell lines correlate with their EMT status, indicated as ratio "EMT" = ((*CDH2*+*VIM*)/2)/*CDH1* (same EMT markers as in Fig. **2a**). Cell lines marked in red were used in Fig. **2a**.

Analysis of CCLE data

Data from the CCLE database were downloaded, normalized and log2 transformed using R software. We defined the EMT level of a cell line as ratio EMT = ((*CDH2*+*VIM*)/2)/*CDH1* of EMT marker expression. Cell lines showing *E2F1* expression and EMT ratio above the median of all cell lines of a distinct tissue type, as well as those with *E2F1* expression and EMT ratio below the median of cell lines of a distinct tissue type were selected, again median centered and normalized and plotted as seen in Supplementary Figure 5.

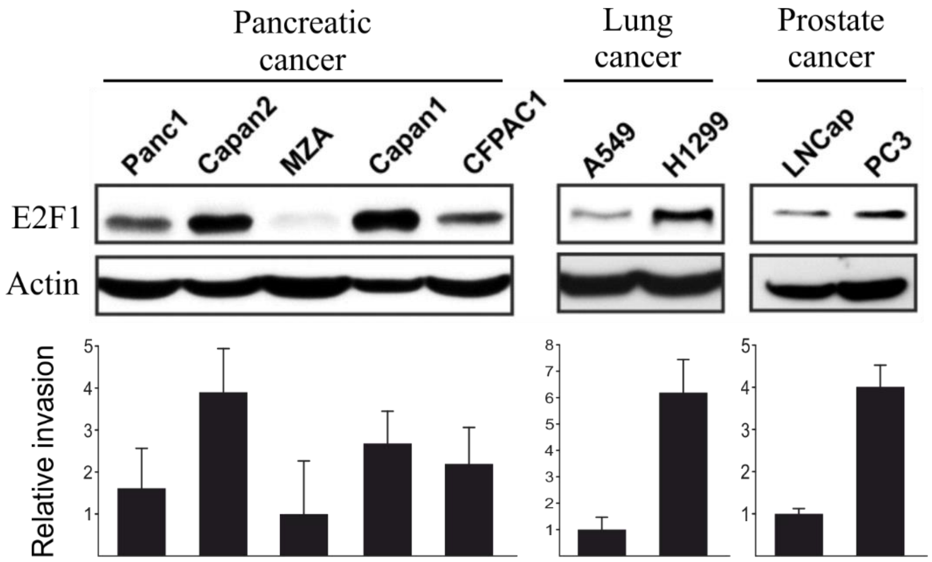

Supplementary Figure 6: Correlation between E2F1 expression and invasiveness in different cancer entities. E2F1 protein levels (upper panel) in association with high vs less invasive growth (bottom panel) of pancreatic, lung and prostate cancer cell lines. Error bars indicate s.d.

Cell culture and treatment

Human pancreatic adenocarcinoma cell lines Panc1, Capan1, Capan2, CFPAC1 (ATCC) and MZA (obtained from D.I. Smith, Mayo Clinic, Rochester, MN, USA), A549, H1299 lung cancer, and LNCap or PC3 prostate cancer cell lines (purchased from ATCC) were maintained at 37°C and 5% CO2 in Dulbecco's modified Eagle's medium (high glucose, 4.5 g per l) containing 2 mM L-glutamine, 1 mM sodium pyruvate, supplemented with 10% FCS, 0.1 mM non-essential amino acids, 50 U per ml Penicillin and 50 µg per ml Streptomycin.

In silico perturbation simulations for the reversal of EMT phenotype

We carried out *in silico* perturbation experiments to identify important nodes that can be exploited for therapeutic interventions. Perturbation experiments were performed for the highly invasive phenotype (EMT=3) by changing Boolean states of each node in the regulatory layer to reduce EMT to a less invasive state (Supplementary Table 4).

Our model simulations predict that in bladder cancer, with a single knockout of either *ZEB1*, *TWIST1*, *SNAI1*, *SMAD2/3/4* or *NFKB1*, or the activation of *CDH1* can bring the phenotype to a less invasive state (EMT=2). Furthermore, simultaneous perturbations: (i) Knockout of *ZEB1* in combination with either *SNAI1*, *TWIST1* or *NFKB1* or with activation of *CDH1*; (ii) knockout of *SMAD2/3/4* in combination with *TWIST1* or *NFKB1* can further reduce the invasiveness (EMT=1). In case of breast cancer, a single knockout of *SRC*, *FN1, SNAI1/2* or the activation of *CDH1* reduces the EMT phenotype (EMT=2). However, double knockout of *SRC*, *FN1, SNAI1/2* or activating *CDH1* in any of the combinations can further reduce invasiveness (EMT=1).

(a) Bladder cancer

E2F1	TGFBR1	FGFR1	ZEB1	TWIST1	SNAI1	NFKB1	SMAD2,3,4	CDH1	EMT
**1**	**1**	**1**	1	1	1	1	1	0	3
**1**	**1**	**1**	0	0	1	1	0	1	1
**1**	**1**	**1**	0	1	0	1	0	1	1
**1**	**1**	**1**	0	0	1	0	0	1	1
**1**	**1**	**1**	0	1	1	1	0	1	1
**1**	**1**	**1**	1	0	1	1	0	1	1
**1**	**1**	**1**	1	0	1	0	0	1	1

(b) Breast cancer

E2F1	TGFBR2	EGFR	SRC	FN1	SNAI1	SNAI2	CDH1	EMT
**1**	**1**	**1**	1	1	1	1	0	3
**1**	**1**	**1**	0	1	1	1	1	1
**1**	**1**	**1**	0	0	1	1	0	1
**1**	**1**	**1**	0	1	0	1	1	1
**1**	**1**	**1**	0	1	1	0	1	1
**1**	**1**	**1**	1	0	1	1	1	1
**1**	**1**	**1**	1	1	0	1	1	1
**1**	**1**	**1**	1	1	1	0	1	1
**1**	**1**	**1**	1	0	0	1	0	1
**1**	**1**	**1**	1	0	1	0	0	1

Supplementary Table 4: Double *in silico* perturbations of highly invasive (EMT=3) phenotype in (a) bladder and (b) breast cancer model. Active state of the molecule is represented by '1', the inactive state by '0'. The first rows in both cancer models represent the predicted molecular signatures for highest EMT level. The dark gray boxes represent the perturbed state of genes and their effect on EMT phenotype is shown in the last column.

Validation of our workflow using predicted and random signatures in TCGA cohorts

We have validated molecular signatures predicted by Boolean simulations in large patient cohorts of TCGA bladder cancer (BLCA; n=426) and TCGA breast cancer (BRCA; n=1218) accessible through UCSC Xena http://xena.ucsc.edu. More precisely, for bladder cancer we selected two subgroups of patients where the individual gene expression of *E2F1*, *TGFBR1* and *FGFR1* was above (signature group) or below (signature* group) the mean expression values, respectively. In case of the breast cancer cohort, similar subgroups were built for *E2F1*, *TGFBR2* and *EGFR* genes. In these subgroups, we identified the mean expression of well-known EMT markers (*CDH1*, *miR-205*, *CDH2*, *VIM*, *SNAI1*, *SNAI2*, *TWIST1* and *ZEB1*) as shown in Supplementary Figure 7. We found that our two signatures were able to distribute patients into early vs advanced stages in bladder cancer and aggressive vs less-aggressive stages in breast cancer significantly (p-value < 0.005).

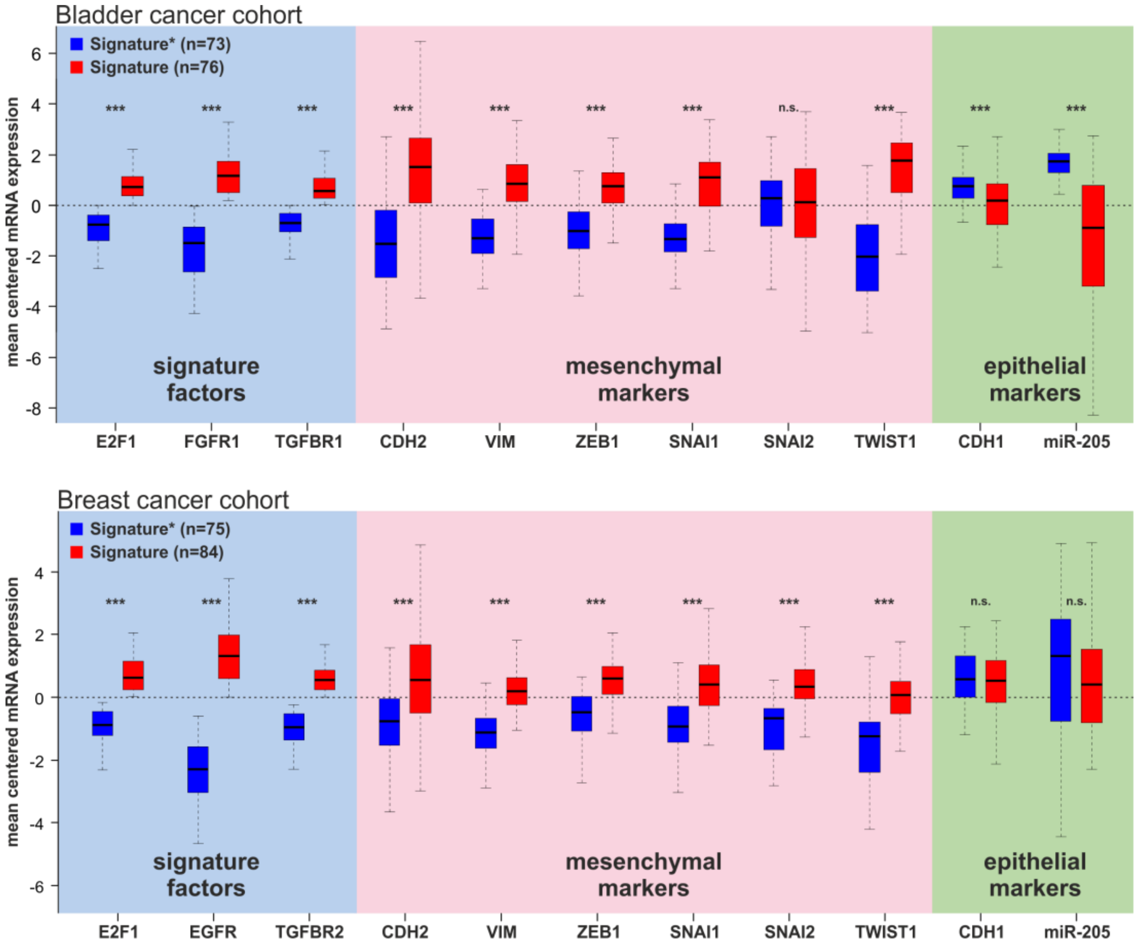

Supplementary Figure 7: Mean centered expression profiles of EMT markers with respect to our molecular signatures in TCGA bladder and breast cancer cohorts. Panels with blue background indicate our molecular signatures, panels with pink background mesenchymal markers and green underlaid panels epithelial markers. Statistical significance was calculated by Student´s t-test (***, p-value < 0.005, n.s. - not significant).

In order to assess the predictive capability of our workflow to find potential molecular signatures, we generated 30 random signatures of three nodes from each of the regulatory cores and arbitrarily assigned high or low expression states with respect to their mean expression value and identified their capability to distinguish patients into clinical stages as mentioned above (Supplementary Data 7 and 8). For each signature and signature* set, we calculated the relative difference of patients in early vs advanced stages in bladder cancer and aggressive vs less-aggressive stages in breast cancer. These differences are plotted in Fig. 8c and 8d.

Comparison of our map with Calzone's map

In the context of our work, Calzone and coauthors reconstructed a comprehensive map of the E2F transcription factor family[10]. However, the Calzone network addresses primarily the role of E2F1 in cell cycle regulation. In fact, the majority of components from Calzone's map are included in our map. We set a main focus on the collection and analysis of data on the basis of new indicators that promote the highly aggressive phenotype of activating members of the E2F family (E2F1-3), with an emphasis on pro- and anti-apoptotic (survival), angiogenic as well as EMT-relevant functions. We included additional key players connected directly to E2F1 or through its neighbors along with a post-transcriptional layer of microRNAs in the context of cancer. In Supplementary Figure 8 we compare the overlaps between ours and Calzone's RB/E2F network.

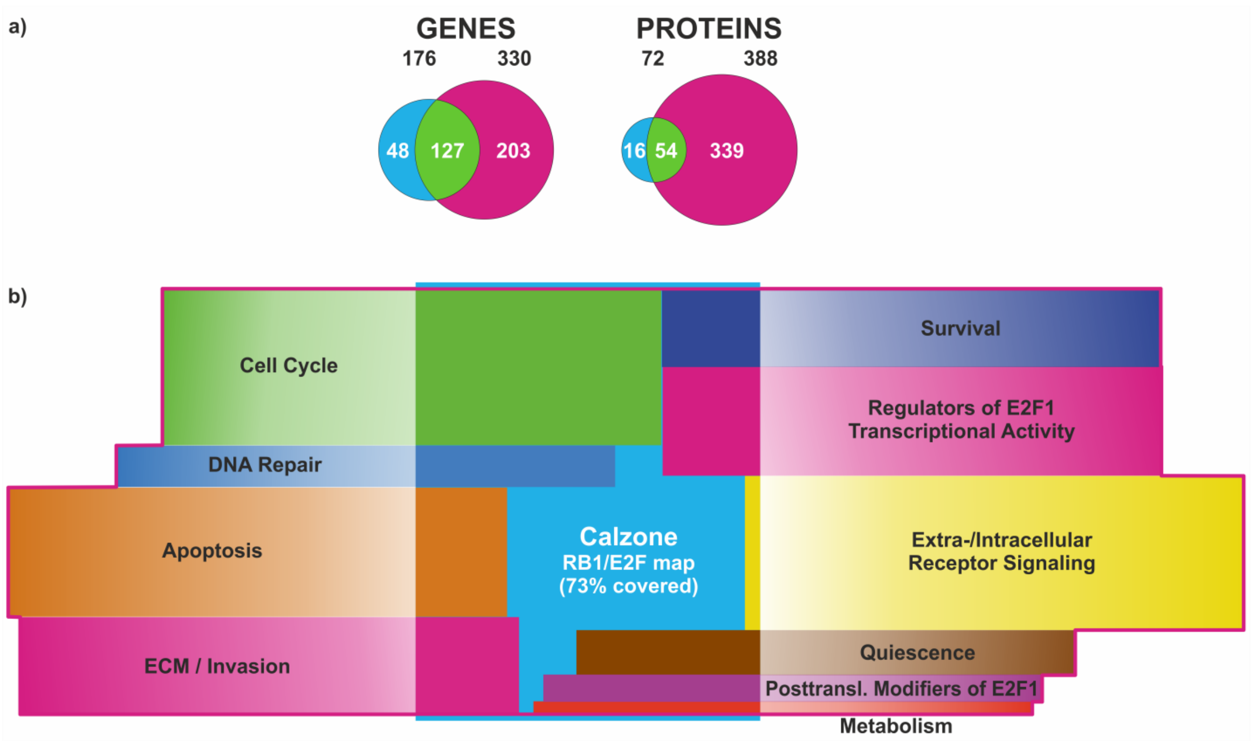

Supplementary Figure 8: Overlaps between our comprehensive E2F1 interaction map with Calzone's RB/E2F network. (a) Numbers and overlaps (green) of genes and proteins between Calzone's (blue) and our map (purple). **(b)** Overlap of the regulatory and functional compartments of our E2F1 map with Calzone's map (light blue bordered box in the center). Diagram indicates that 73% of the components of Calzone's RB1/E2F map are included in our E2F1 map. Our map is almost 2.8 times bigger than Calzone's map.

Fig.2a

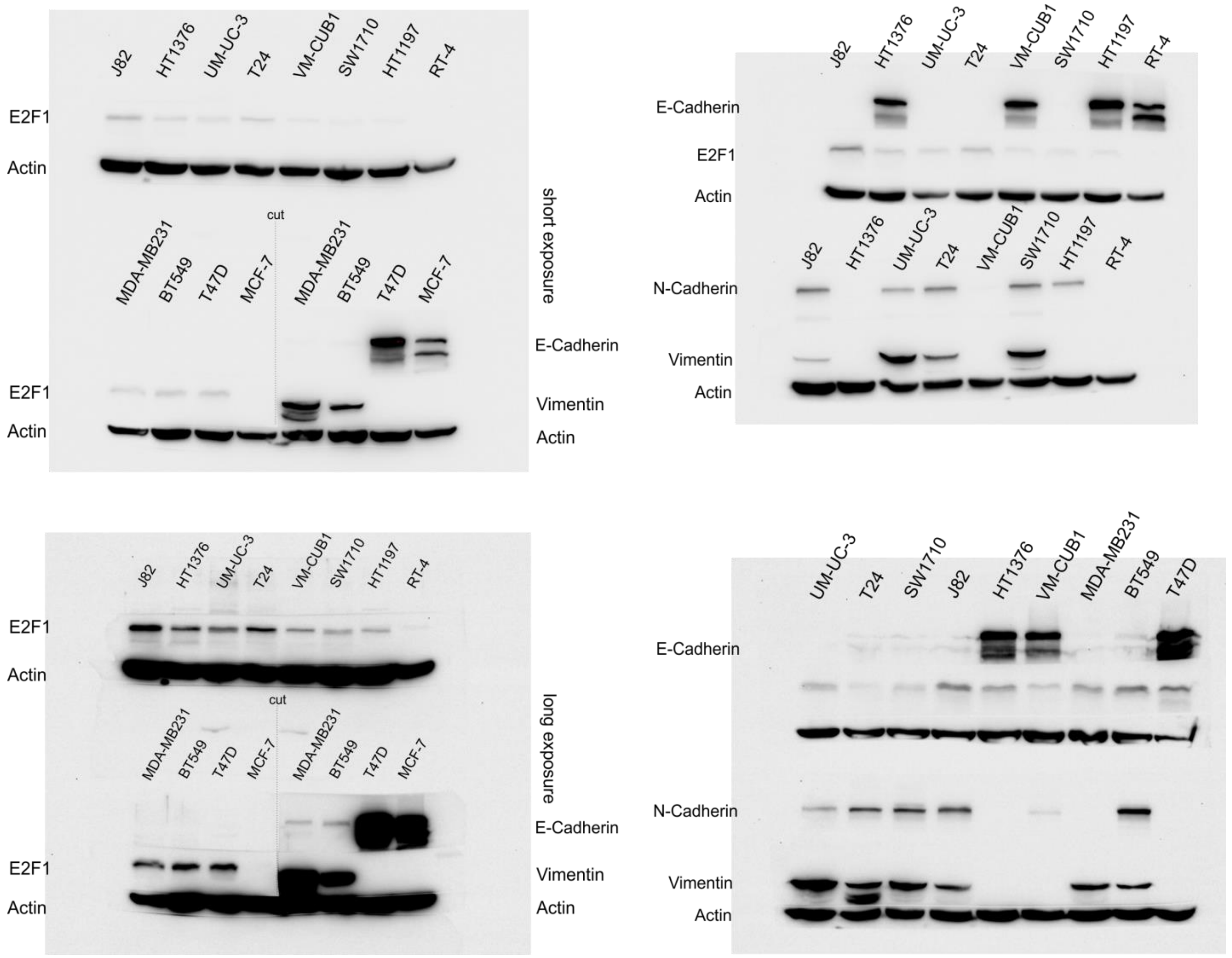

Supplementary Figure 9: Uncropped pictures of the Western blots shown in the indicated figure.

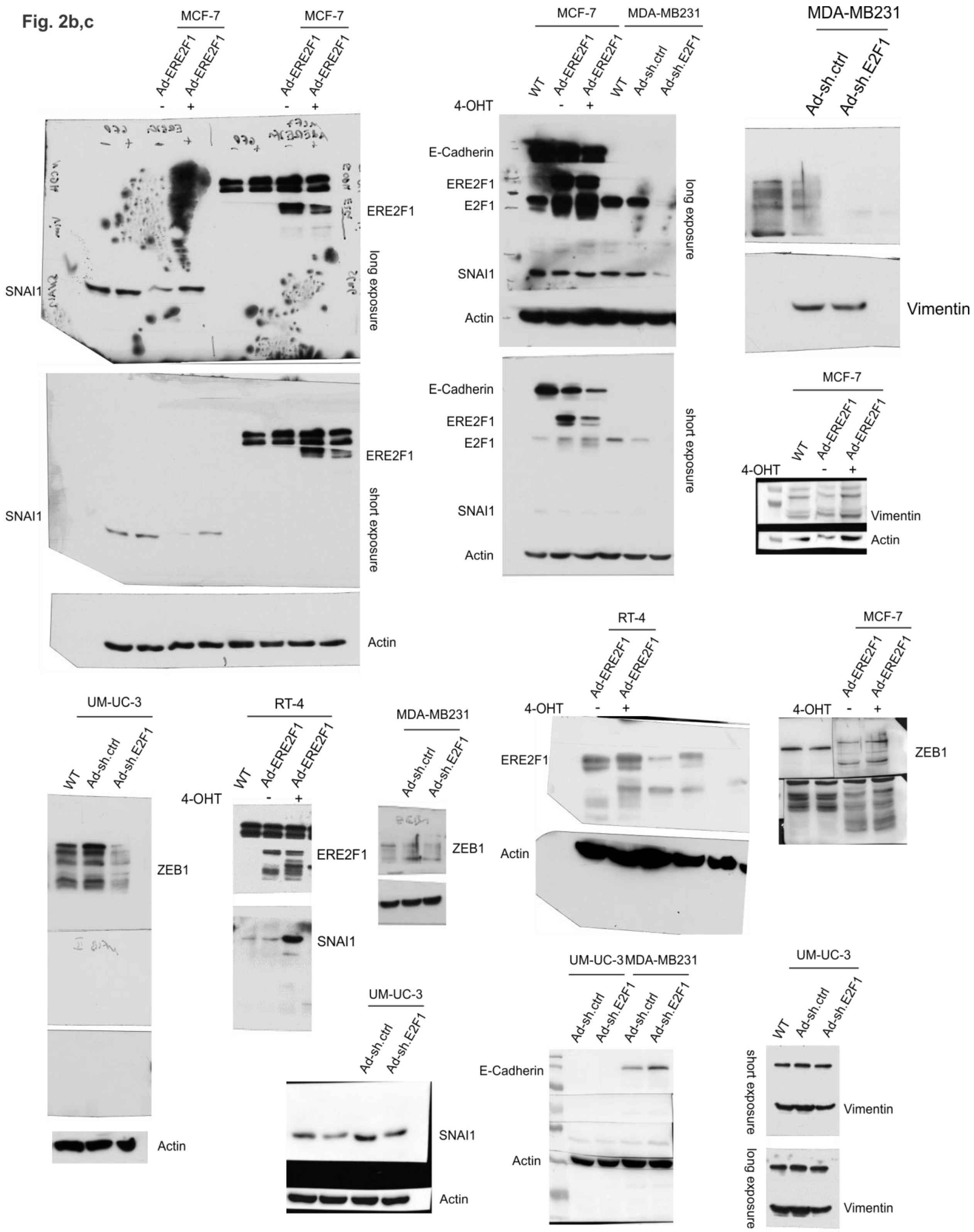

Supplementary Figure 10: Uncropped pictures of the Western blots shown in the indicated figures.

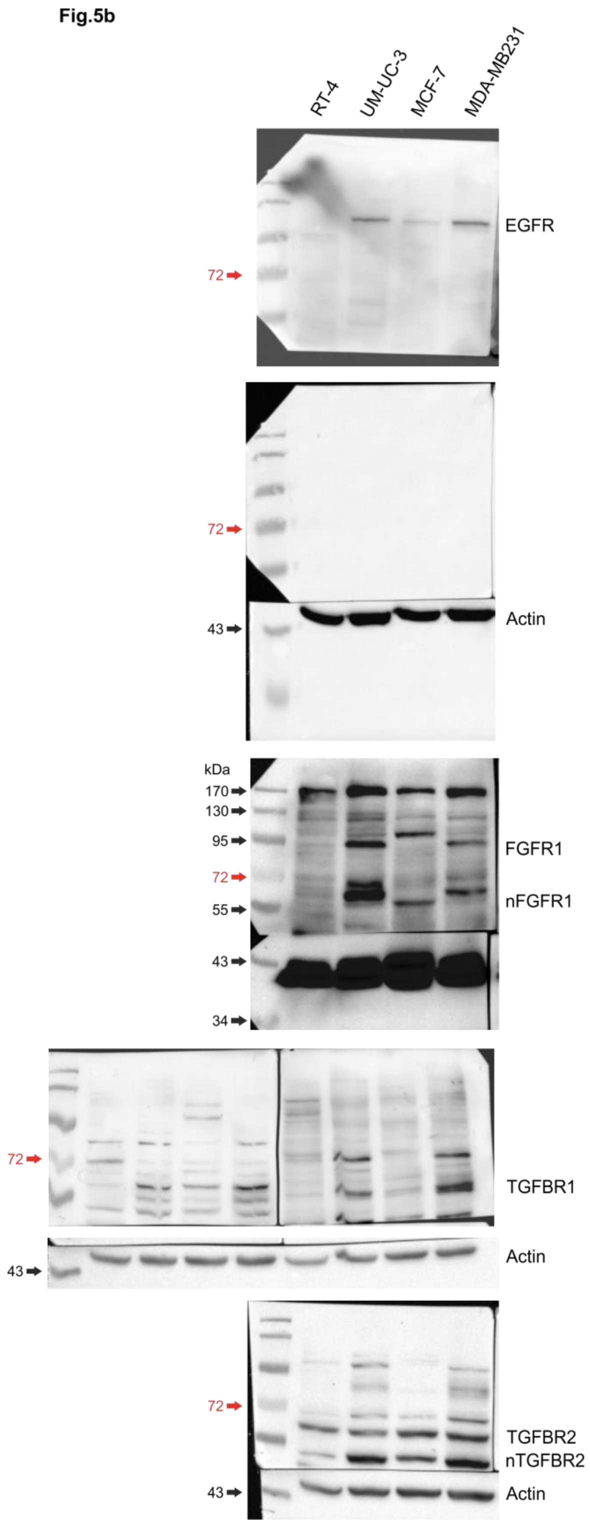

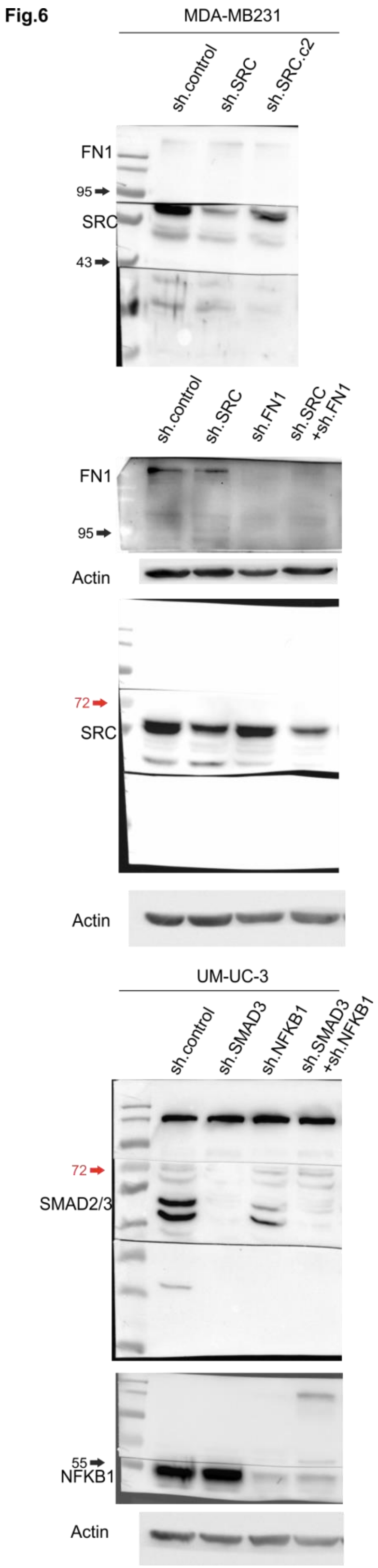

Supplementary Figure 11: Uncropped pictures of the Western blots shown in the indicated figures.

Supplementary References

1. Shannon, P. *et al.* Cytoscape: A software Environment for integrated models of biomolecular interaction networks. *Genome Res.* **13,** 2498–2504 (2003).
2. Watts, D. J. & Strogatz, S. H. Collective dynamics of 'small-world' networks. *Nature* **393,** 440–442 (1998).
3. Zhang, Z. & Zhang, J. A big world inside small-world networks. *PLoS One* **4,** e5686 (2009).
4. Ravasz, E., Somera, A. L., Mongru, D. A., Oltvai, Z. N. & Barabási, A. L. Hierarchical organization of modularity in metabolic networks. *Science* **297,** 1551–1555 (2002).
5. Barabási, A.-L. & Oltvai, Z. N. Network biology: understanding the cell's functional organization. *Nat. Rev. Genet.* **5,** 101–113 (2004).
6. Fell, D. A. & Wagner, A. The small world of metabolism. *Nat. Biotechnol.* **18,** 1121–1122 (2000).
7. Peng, Q. & Schork, N. J. Utility of network integrity methods in therapeutic target identification. *Front. Genet.* **5,** 12 (2014).
8. Albert, R., Jeong, H. & Barabási, A. L. Error and attack tolerance of complex networks. *Nature* **406,** 378–382 (2000).
9. Steinway, S. N. *et al.* Network modeling of TGFB1 signaling in hepatocellular carcinoma epithelial-to-mesenchymal transition reveals joint sonic hedgehog and Wnt pathway activation. *Cancer Res.* **74,** 5963–5977 (2014).
10. Calzone, L., Gelay, A., Zinovyev, A., Radvanyi, F. & Barillot, E. A comprehensive modular map of molecular interactions in RB/E2F pathway. *Mol. Syst. Biol.* **4,** 173 (2008).
